# Developing the Fear of Disasters Scale and Exploring Its Psychometric Properties

**DOI:** 10.1155/2024/5565403

**Published:** 2024-11-25

**Authors:** Aysun Güzel

**Affiliations:** Emergency Aid and Disaster Management, Faculty of Health Sciences, Burdur Mehmet Akif Ersoy University, Burdur, Türkiye

**Keywords:** community, disasters, fear, psychometric property, Turkey

## Abstract

**Aims:** The present methodological study attempted to develop the Fear of Disasters Scale (FDS) and reveal its psychometric properties.

**Methods:** While explanatory factor analysis (EFA) was carried out on the data of 265 voluntary participants, the data of 75 participants were used to explore the test–retest reliability of the scale. Confirmatory factor analysis (CFA) was carried out on the data of 150 participants. All analyses were performed on the Statistical Package for the Social Sciences (SPSS) 25.0 and linear structural relations (LISRELs) programs.

**Results:** Cronbach's *α* value was calculated to be 0.93. The test–retest reliability analysis resulted in a significant, very strong, and positive correlation (*p* < 0.001; *r* = 0.92). The CFA yielded the following fit indices for the scale: *p* > 0.05, *χ*^2^/df = 1.51, root mean square error of approximation (RMSEA) = 0.05, standardized root mean square residual (SRMR) = 0.02, goodness of fit index (GFI) = 0.96, normed fit index (NFI) = 0.97.

**Conclusions:** The findings revealed that the one-factor instrument with seven items is valid and reliable for measuring fear of disasters (FD) in the sample.

## 1. Introduction

Disasters, defined as the destructive impacts of natural or human-made forces exceeding the resilience capacity of a particular region or community, always cause unexpected deaths, injuries, job losses, and material damages [1]. The calculation of the exposure and vulnerability of people to the hazards that constitute disasters is called “disaster risk” [2]. Turkey's geological and geomorphological structures mediate frequent natural and technological disasters [3]. In addition to being among the regions most affected by earthquakes [4], it has been dramatically affected by other disasters such as global warming, climate changes, forest fires, desertification, and drought in recent years [5]. In 2019 reports, it is stated that Burdur province is risky in terms of disasters such as earthquakes, floods, and landslides [6] and has a high seismic risk (The World [7]). It is emphasized in the literature that Burdur province is also affected by many natural and man-made disasters such as fire, explosion, extreme winter conditions, storms, traffic accidents, and terrorism [8]. It should be noted that the impacts aggrege in cities since the rapid increase in population causes unreliable urbanization. Loss of life and property due to disasters increases within the population with increased vulnerability [9]. In addition to increased incidents [10] and situations, such as being vulnerable to disasters or not being able to cope with them quickly, may cause distress, apprehension, and fear among people [11]. In particular, uncertainties about a disaster and how it will affect one's family and oneself may drive people to experience a deep fear [12].

Fear is a feeling emerging in case of danger or the expectation of danger. By its nature, it is considered among the oldest and strongest feelings of humanity [13]. The fear of disaster, on the other hand, is highly associated with the awareness of disasters, risk perception, uncertainty, ability to control events, optimism bias towards the events, and trust in people [14]. Death of family members or close relatives, loss of property, work-related disruptions, and disruptions in local services and infrastructure are common situations during disasters, and thinking of the possibility of such conditions may lead to fear in people [15]. Besides, the incidents of theft [16], increased frequency of domestic violence and criminal cases [17], and evacuation and forced migration [18] in disasters may also elevate the fear of disasters (FD).

The relevant literature hosts many studies on the FD. The common aspects of the previous research were to attempt to identify the FD among people using several measurement tools [19–21]. The findings often highlighted that females have more FD than males and that the parents are more concerned about their children than themselves [19]. In general, FD mostly stems from volcanic eruptions [20] and natural disasters caused by floods [21]. Some other studies touched upon the effects of disasters on mental health, especially fear-related behavior [22, 23].

For those indirectly exposed to disasters, fear—especially FD—becomes a prerequisite to keep themselves safe and take action in crises [19]. For those experiencing disasters, fear causes vulnerability, shame, anger, tears, and sadness, as well as triggering some other fears (e.g., fear of animals, the dark, and imaginary events). In both cases, the FD is likely to affect one's life and leads them to worry much [24]. Yet, such fear and worries may initiate positive behavior for disaster preparedness [19]. It may also change one's values and/or behavioral tendencies. Interestingly, people tend to improve their social welfare in communities with prevalent FD [25].

Measuring the FD is deemed important for promoting disaster perception and awareness within society. Therefore, exploring the FD may become essential to motivate and develop one's disaster preparedness and generate communication and guidance networks [19]. Nevertheless, the relevant literature lacks an instrument to measure the FD. Ultimately, the present study attempted to answer the following research question:

Is the Fear of Disasters Scale (FDS) a valid and reliable instrument in the Turkish population?

## 2. Materials and Methods

### 2.1. Purpose and Design

The present methodological study aimed to develop the FDS and explore its psychometric properties.

### 2.2. Development of Item Pool

Prior to engaging in the development phases, the researcher sought answers to some essential questions (e.g., Is there a need for a new scale? What does the scale aim to measure? Who will the scale be developed for? How will the scale be administered? Is the implementation period of the scale important?) and then, addressed the conceptual framework of the scale to be developed. In the literature, there is no specific measurement tool for disaster fear. Moreover, a similar structure to the intended scale has not been conceptually defined before. The scale was developed to measure the fear toward disasters at any time in daily life. Even though there are individual-centered practices oriented to disaster preparedness and education and determining and alleviating fear before or after a disaster, they should include the whole society. Therefore, it was decided to design a measurement tool oriented to those over the age of 20, standardized in terms of the conditions where the scale would be administered to the target population (the scale should be administered under supervision to people in streets, squares, parks, workplaces, and cafes), and featured as a self-report instrument since the sample would consist of the general population. Moreover, it was attempted to ensure the statements on the scale and administration time were not too long.

When creating the item pool for the FDS, the definition of the term “fear” was first investigated. By definition, fear is an emotion experienced in anticipation of some threat or danger. Although there is no exact definition of disaster fear in the relevant literature, it is emphasized that it is influenced by past experiences (perception of danger and risk is high in disaster experiences, thus, disaster fear is also high) [21]. It is often emphasized in the literature that the conceptual definition of psychological variables is a significant and rigorous issue [26]. Thus, it may be proposed that disaster fear is a state with individual differences and may change depending on how the danger is experienced.

After settling on a conceptual definition of disaster fear, the researcher reviewed the previous work on pre-, mid-, and postdisaster fear in the literature (the introduction section addresses the relevant studies). Scales utilized in these studies to measure fear, scale items, and the scientific background of these items were also examined. Then, the variables of the newly developed scale were determined in light of the items reviewed and the definition brought to disaster fear.

While creating the statements, the researcher considered the characteristics of the target population (age group and gender), the changes in the environment in which the scale was intended to be administered, culture, and the adaptation of the indicators in the available measurement tools to the FDS. While developing the scale, the researcher all complied with all of the criteria claimed to be among the indicators of the conceptual definition: bringing a definition to the concept, identifying relevant indicators, pooling the items, and evaluating whether the scale has a correspondence in daily life, can be responded independently of the environment, and is suitable for the target group [26]. The instrument intended to be created in this study aims to measure fear. In a study in the literature, during the assessment of FD, the level of concern for oneself, one's life and others during earthquakes, floods or epidemics was asked, and the respondent was asked to score between one and five. In the “disaster perception model” created in the same study, disaster fear was defined as the fear or anxiety felt against any disaster (earthquake, flood, tsunami, etc.) [27]. In this context, the scale consists of statements oriented to the fear felt when thinking of disasters. The statements are presented in the findings section.

### 2.3. Content Validity

Regarding content-related validity of the FDS, the relevant literature was first examined, and an item pool of 25 items was generated [19–21, 23]. After a public health specialist and a psychiatrist went through the items, inconvenient, replicated, and unclear items were removed from the data set, and the remaining 17 items were considered for the analyses. The removed items were “I am afraid of being exposed to,” “I am afraid that a family member of mine will be exposed to,” “I am afraid that the health of others around will be adversely affected,” “I am afraid that job continuity (inability of public or private sector employes to continue their jobs) will be adversely affected,” “I am afraid that the health of street animals will be adversely affected,” “I am afraid that the environment (cultural values, urban infrastructure) will be damaged,” “I am afraid that the search and rescue teams cannot reach me,” and “I am afraid of not being able to benefit from the financial resources allocated by the state.”

Furthermore, to obtain expert opinions, the draft form was submitted to a total of 16 specialists via e-mail. They were asked to assess the suitability of the items and tick one of the options on an evaluation form [28]. Upon the feedback from 10 specialists, the content validity ratio (CVR) index and content validity index (CVI) were calculated for each of the 17 items.

### 2.4. Sample

The data were collected three times separately in this study. While initial data were collected for explanatory factor analysis (EFA), the others were collected to perform test–retest reliability analysis and confirmatory factor analysis (CFA), respectively. All the data were collected from people aged 20–69 years living in Burdur city of Turkey. For the first and third sampling procedures, it was considered that 20 times the number of items should be reached [29]. However, different calculations are suggested to determine the sample size in validity and reliability studies. In a study in the literature, it is recommended that at least 10 people be included in the sample for each item in the scale. In the same study, it is emphasized that a sample of 300 people is “good” and a sample of 500 people is “excellent” and working with a large sample can help to ensure the stability of factor analysis [30]. In this study, some items in the draft measurement tool were removed after the CVR and CVI analyses. Immediately afterwards, some other items that were highly correlated with each other were also removed and 12 items remained in the draft measurement tool for the first sample. Therefore, a sample of 300 participants was considered to be appropriate for the first sample. In the first sampling, it was aimed to work on the data of 300 people recruited from the target population, 60 from each age group specified (20–29, 30−39, 40–49, 50−59, and 60–69 years). Nevertheless, the Corona Virus Disease 2019 (COVID-19) pandemic, the difficulty in reaching people aged 60–69 years, and the consideration of the second phase of the research eventually caused the researcher to reach only 53 people in the last group. Hence, the first sample was composed of 265 people, 53 people in each age group. In the literature, it is emphasized that in the test–retest phase, it is appropriate to reach 25% of the participants in the first sample [28]. Therefore, 75 people were considered appropriate for the second sample. The second sample was composed of 75 people resampled from the main sample after 2 weeks (15 randomly selected individuals from each age group). After the exploratory factor analyses, a total of seven items remained in the measurement tool and it was considered appropriate to reach 150 people within the scope of the literature. Following the data collection and analysis in the first and second samples, the third sample consisted of 150 people resampled from the main sample (30 randomly selected people from each age group).

In this study, quota sampling, among the nonprobability sampling methods, was used to recruit the participants (for all three samples). The participants were included in the study by age group. The researcher included only those living in Burdur city, being 20–69 years, and being capable of understanding the items and expressing their thought

The first and second data were collected between May 15 and August 15, 2021, while the third data were collected between May 9 and 31, 2022 in the streets, squares, parks, workplaces, and cafes in the city center. Data collection took 20–25 min per participant.

### 2.5. Data Collection Tools

The researcher collected the data using a booklet covering a demographic information form (10 items), the Fear of COVID-19 Scale (FCS; 5 items), and the FDS (12 items for the first and second samples and 7 items for the third sample).

Outbreaks such as COVID-19, which affect the entire society, cause a sudden increase in the number of patients and inadequate health services, are considered as “disasters” [31]. The COVID-19 pandemic has negatively affected people's physical health and lives. In addition to physical health, it also caused mental health problems such as stress, anxiety, and depression by causing fear and panic in the society [32]. For these reasons, the FCS, which covers both one of the disaster types and assesses the fear of this disaster, was used in this study. The FCS was developed by Ahorsu et al. [33] and adapted into Turkish by Bakioğlu, Korkamaz, and Ercan [32]. It is a single-factor scale with seven items and has no reverse coded item. One may score between 7 and 35 points on the scale, and high scores indicate experiencing a high level of fear of coronavirus [32]. While Ahorsu et al. [33] calculated Cronbach's *α* value to be 0.82, Bakioğlu, Korkamaz, and Ercan [32] found it to be 0.88.

The FDS, psychometric properties of which were explored in this study, was developed utilizing various sources [19–21, 23]. The steps followed to create the scale were as follows: generating an item pool, designing the measurement method (6-point Likert-type scale ranging from “0” (No fear) to “5” (Extreme fear), obtaining expert opinions for the draft form, and calculating CVR and CVI, finalizing the draft form as a scale consisting of seven items with instructions.

### 2.6. Data Analysis

For the first sample, Statistical Package for the Social Sciences (SPSS) 25 was used to perform validity (CVR and CVI, exploratory factor analysis, and convergent validity) and reliability (item analysis and internal consistency reliability) analyses. The same program was used to perform a test–retest reliability analysis for the second sample. Finally, CFA was performed on linear structural relations (LISRELs) for the third sample.

## 3. Results

### 3.1. Sample Characteristics

For the first sample, the mean age of the participants was 42.3 (standard deviation (SD) = 13.5). About half of the participants (51.3%) were males; 41.9% had an undergraduate degree; 50.9% were married; 44.5% reported high socioeconomic status. Since the second sample was composed of those randomly selected among those in the main sample, their demographic characteristics were not restated. Regarding the third sample, the mean age of the participants was 42.1 (SD = 13.4). About half of the participants (51.3%) were males; 43.3% had an undergraduate degree; 44.0% were married; 48.7% reported high socioeconomic status (Table 1).

Except for the test–retest phase (second sample) and CFA analysis (third sample), all validity and reliability analyses in the study were performed on the first sample.

The singularity and multicollinearity issues were investigated on the FDS before performing validity and reliability analyses (except for CVR and CVI), and no singularity problem was detected in the items. Nevertheless, the following items yielded high correlations (*r* > 0.80 or *r* > 0.90) with each other and other items: “I am afraid the health of my second-degree relatives (brother, grandchild, grandfather, grandmother, grandmother) will be adversely affected,” “I am afraid that the health of my spouse (or prospective spouse) will be adversely affected,” “I am afraid that the health of special interest groups (children, the disabled, or older adults) will be adversely affected,” “I am afraid of being deprived of food,” and “I am afraid that I will not have a place to stay.” After these items were excluded from the analysis, only seven items remained on the FDS.

### 3.2. Validity

CVR and CVI were first calculated to explore the validity of the FDS. Following the analyses, it was found that an item was excluded from the dataset since yielding zero (0) or negative (less than zero) CVR value (“I am afraid of not being able to reach AFAD (Disaster and Emergency Management), UMKE (National Medical Rescue Teams), or 112 Emergency Hotline”). Besides, the researcher also removed two items with a CVR value below 0.62 (“I am afraid of secondary hazards (fire, landslide, etc.)” and “I am afraid of not being able to access basic healthcare services (first aid, emergency aid, etc.)”) and two items with contradictory and negative comments by the experts (“I am afraid that my house will be damaged” and “I am afraid that the things in my house will be damaged”). In line with the suggestions of the experts, the phrase “damage to my house or the things in my house” was added to the item “I am afraid of economic harm.” Moreover, the item “I am afraid that the health of my first-degree relatives will be adversely affected” was expanded with “mother, father, and children” in parenthesis. Finally, the item “I am afraid that the health of my second-degree relatives will be adversely affected” was expanded with “sister, grandchild, grandfather, grandmother, grandmother” in parenthesis. Besides, the mean CVI value of the remaining 12 items was found to be 0.84; hence, the scale items were statistically significant.

After calculating the CVR and CVI values of the items, 12 items remained in the measurement tool. Moreover, five more items were excluded from the dataset due to multicollinearity. Thus, EFA analyses were performed on the remaining seven items. The Kaiser–Meyer–Olkin (KMO) test revealed the sample adequacy to be 0.925; thus, the sample size of 265 people was concluded to be sufficient for EFA. As a result of Bartlett's test of sphericity, the chi-square value (*χ*^2^_(21)_ = 1476.178; *p* < 0.001) was found to be statistically significant. Following satisfying the assumptions, the EFA resulted in a one-factor structure with seven items explaining 73.23% of the total variance. The item factor loadings were found to be the lowest with 0.83 in item 5 and the highest with 0.88 in item 6 (Table 2).

**Figure 1:**
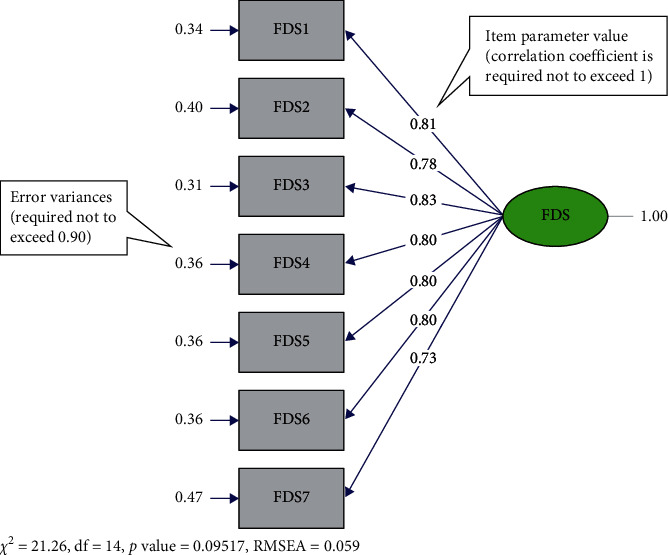
Standardized analysis—CFA. CFA, Confirmatory Factor Analysis; FDS, Fear of Disasters Scale.

**Figure 2:**
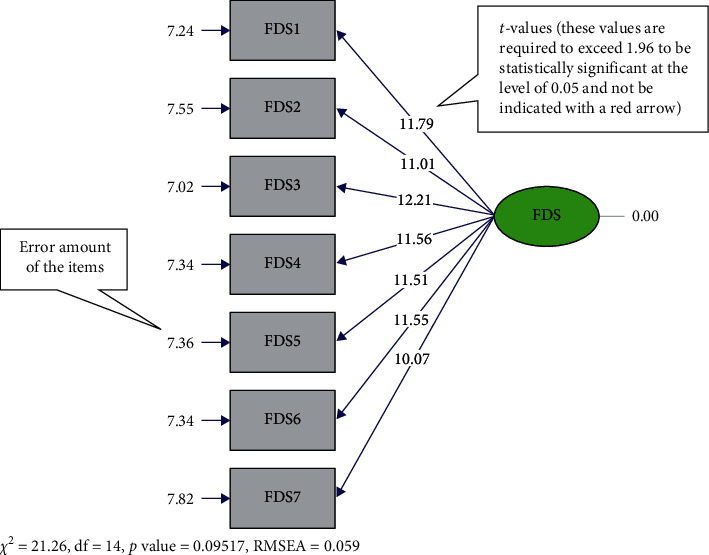
*t*-values—CFA. CFA, Confirmatory Factor Analysis; FDS, Fear of Disasters Scale.

The factor structure of the variables was revealed through the EFA performed with the data collected from the first sample. CFA, on the other hand, was performed on the data from a different sample. The purpose of performing CFA is to investigate the compatibility between the factors revealed in EFA and the factors suggested theoretically. In the CFA, standardized regression weights, *t*-values, and error variances of the items were explored. Accordingly, the CFA yielded no item with an error variance above 0.90. The lowest and highest error values were found to be 0.31 in item 3 and 0.47 in item 7, respectively (Figure 1). It was also found that the standardized regression weights of the items ranged between 0.73 and 0.83. Moreover, there was no item with a *t*-value below 1.96 and indicated with a red arrow. While the lowest *t*-value was found to be 10.07 in item 7, the highest value was found to be 12.21 in item 3 (Figure 2).

The CFA results yielded the following fit indices for the FDS: *p* > 0.05 (*p*=0.095), *χ*^2^/df = 1.51, root mean square error of approximation (RMSEA) = 0.05, root mean square residual (RMR) = 0.07, standardized RMR (SRMR) = 0.02, goodness of fit index (GFI) = 0.96, normed fit index (NFI) = 0.97, CFI = 0.99, incremental fit index (IFI) = 0.99, and expected cross validation index (ECVI) = 0.33 (Table 3). Hence, it can be confidently asserted that the conceptual model of the 7-item FD showed acceptable fit to the data.

Finally, convergent validity of the FDS was investigated with the help of the FCS. Accordingly, a significant, weak, and positive correlation was found between the FCS and FDS total scores (*p*  < 0.001; *r* = 0.31; Table 4).

### 3.3. Reliability

The reliability of the FDS was examined by exploring item-total correlations, internal consistency reliability, and test–retest reliability of the scale on the SPSS 25.0 program.

The lowest item-total correlation was computed to be 0.76 in item 5, while the highest was 0.83 in item 6. Besides, Cronbach's *α* value was calculated to be 0.93 for the total score (Table 5). Finally, the test–retest reliability analysis resulted in a significant, very strong, and positive correlation (*p* < 0.001; *r* = 0.92).

## 4. Discussion

The present methodological study aimed to develop an instrument to measure the FD among individuals aged 20–69 years.

Preparing for disasters is shaped by risk perception, previous disaster experiences, the level of exposure to disasters, socioeconomic and psychological factors, and FD [37]. Disasters may exert direct and indirect impacts on individuals. While direct impacts are evident in disaster survivors, indirect influences are observed in relatives of disaster victims, rescue workers, or those following these events on social media [38]. The fundamental mechanism of fear is triggered by danger. Similarly, the psychological problems occurring during disasters are strongly associated with the subjective perception of danger [39]. Identifying one's perception of danger, and therefore, fear is considered important to designate public education and risk communication. Effective risk communication may not only alleviate fear but also contribute to disaster preparedness in society by promoting self-protective behavior, building trust, and preventing the spread of misinformation [40]. Furthermore, measuring the FD may offer fruitful information to promote disaster preparedness, to design mental health education and leadership, team, and peer training, and to identify vulnerable groups needing additional support. Besides, enabling society to be able to cope with disasters is only possible through activities such as workshops on emotional/psychological well-being and social supports to increase resilience. Identifying the FD among those exposed to disasters both directly and indirectly may be necessary to reinforce disaster awareness and preparedness [39].

The risk perception of disasters and FD are affected by social stratification, inequality, and power and vary by vulnerability to disasters, social roles, and gender inequality. The social roles particularly influence the risk perception and the level of fear. For example, women tend to be more fearful and risk-avoidant, unlike men. Hence, women become more volunteer, active, and willing to receive training for disaster preparedness. This and similar differences may imply that perceived risk, fear, and disaster preparedness may exhibit substantial changes among people; therefore, identifying the presence and level of fear may have an initiating role in disaster preparedness [41, 42]. In this study (all samples), approximately half of the participants were women, and the researcher consciously tended to designate equal distribution and representation of age groups in the study. Moreover, the sample was selected from the general population since it is only possible to establish disaster preparedness with the contributions of each citizen in society.

As part of the development of the FDS, the researcher first prepared an item pool consisting of 25 items. The item pool was then submitted to a public health specialist and a psychiatrist to evaluate the items, resulting in the draft form of FDS with 17 items. The draft form was next presented to 10 academics in various fields for obtaining expert opinions. The literature often recommends that the number of experts evaluating Likert-type scales should be between 5 and 40 and that the smallest CVR value of an item evaluated by 10 experts should be greater than 0.62 (*p* < 0.05) [28]. CVR is an item statistics regarding the content validity of items and calculated with a formula covering the number of experts accepting the item as appropriate and the total number of experts giving their opinions on the item. The CVI, on the other hand, is the mean value of the CVR values of the items decided to be included in the scale [43]. Following CVR and CVI calculations, five more items were removed from the scale, and 12 items remained on the scale. Moreover, some items were corrected upon further comments by the experts. The CVI value of the final draft was found to be 0.84, which is greater than the cutoff value recommended in the literature (0.67) [28]. It is recommended that the final scale may consist of 20–25 items in Likert-type scales, and therefore, several times of 20–25 items should be written in the first item pool [26]. In this study, the number of experts evaluating the scale and the CVI value calculated for the 12-item final draft were found to be compatible with the literature. However, the number of items in the item pool created was less than the one suggested in the literature, which was because the items were pooled in line with the relevant literature presenting a restricted insight into the subject.

It is essential to ensure the suitability of the database for factor analyses [44]. Thus, the singularity and multicollinearity issues of the items were also investigated prior to the factor analyses. While there was no singularity problem, some items yielded high correlations with each other and other items. It is often stated in the literature that multicollinearity between the items *r* > 0.80 [45] or >0.90 [46] should be minded before the factor analyses [47]. In this study, five items were found to be highly correlated with each other and other items and removed from the database prior to EFA.

The results of the EFA revealed the factor loadings of the items to range from 0.83 to 0.88 and a one-factor structure explaining 73.23% of the total variance. In this study, there were no items with a factor loading below the cutoff value of 0.32 [44]. The variance explained by the resulting one-factor structure and the factor loadings of the items were both consistent with the literature [44]. The literature generally indicates the variance explained in factor analysis should be between 40% and 60% for social sciences [44] or that it should provide at least 60% [48].

Then, the researcher sought convergent validity. Convergent validity is provided by administering a newly developed scale simultaneously with a previously designed scale with proven validity and reliability and calculating the correlations between the two scales [49]. As expected, a significant positive correlation was found between the FCS and the FDS (*r* = 0.31). The FCS consists of seven items covering discomfort, worry, and physical changes to explain one's fear of COVID-19 [33].

On the other hand, Cronbach's *α* values and item-total correlations were examined to put forward evidence for the reliability of the FDS. The lowest item-total correlation was found to be 0.76 in item 5, and all item-total correlations were above 0.25 [50]. Moreover, the Cronbach's *α* value was calculated to be above 0.70 (*r* = 0.92), accepted as the cutoff value in the literature [49].

Test–retest reliability of the scale was analyzed as the final step of reliability analyses. The results revealed a high (above 0.90) correlation between the test–retest FDS total scores, which is consistent with the limit values suggested in the literature (0.90–1.00 = very strong correlation) [28].

CFA was performed, and path diagrams were examined in the second step of the validity analyses. It is generally accepted that error variances should not exceed 0.90 [35] and that the standardized regression weights of the items should be 0.50 and above [51]. In line with the literature, there was no item with an error variance above 0.90 and standardized weight below 0.50 (Figure 1). Also, *t*-values of the items are required to exceed 1.96, be statistically significant at the level of 0.05, and not be indicated with red arrows by the program [35] (Figure 2). In this study, no item was found with a *t*-value less than 1.96.

Concerning fit indices, the literature often cites CFI, RMSEA, RMR, SRMR [34, 36], NFI, and non-NFI (NNFI) [36] as well as *χ*^2^/df. In this study, the suggested modifications were performed to improve the goodness-of-fit indices. Following modifications, the CFA yielded the following fit statistics:*p* > 0.05; *χ*^2^/df = 1.51; RMSEA = 0.05; SRMR = 0.02; GFI = 0.96, NFI = 0.97; NNFI = 0.98; CFI = 0.99; and IFI = 0.99) [34, 36]. Hence, it can be confidently asserted that the conceptual model of the seven-item FD showed an acceptable fit to the data.

### 4.1. Limitations of the Study

The present study is not free from a few limitations. First, the newly developed FDS was administered to only a sample selected from Turkey. Thus, further research may consider examining its psychometric properties in different cultural contexts. Second, there was no control item (negative statement) on the scale. Third, it was assumed that those participating were able to fill out the questionnaire appropriately.

## 5. Conclusion

In this study, it was explored whether FDS validly and reliably measures FD. Accordingly, the researcher sought validity (expert opinions, CVR, CVI, EFA, convergent validity, and CFA) and reliability (item statistics, internal consistency (Cronbach's *α*), and test–retest reliability) of the scale. After calculating CVR and CVI values, five items were removed from the initial 17-item draft, and the data were collected with the remaining 12 items. The multicollinearity problem led the researcher to delete five more items from the dataset.

In this study, the psychometric properties of the FDS were investigated. The findings revealed that the one-factor instrument with seven items is valid and reliable for measuring FD in the sample group (Turkey/Burdur city, general population, individuals aged 20–69 years).

The FDS may be a useful tool in uncovering the FD among individuals, which may be key for stimulating disaster awareness and preparedness in society and organizing psychoeducation and volunteer programs for professionals. It may also help ensure disaster mitigation efforts to be sustainable.

## Figures and Tables

**Table 1:** Distribution of participants in the study according to various sociodemographic characteristics.

Characteristics of Participants^a^	First sample (*n* = 265)		Third sample (*n* = 150)	
	*n*	%	*n*	%
Sex				
Male	136	51.3	77	51.3
Female	129	48.7	73	48.7
Marital status				
Married	135	50.9	66	44.0
Single	120	45.2	66	44.0
Widowed	10	3.9	18	12.0
Educational status				
Primary school	19	7.2	2	1.7
Secondary school	44	16.5	28	18.4
High school	91	34.4	55	36.6
University	111	41.9	65	43.3
Economic status				
Poor	50	19.0	27	17.6
Undecided	97	36.5	50	33.7
Good	118	44.5	73	48.7
Chronic disease status				
Yes	112	42.4	60	40.2
No	153	57.6	90	59.8
Continuous medication status				
Yes	55	20.7	29	19.2
No	210	79.3	121	80.8

^a^Since the second sample was composed of those randomly selected among those in the main sample, their demographic characteristics were not restated.

**Table 2:** Factor loadings of the items.

	Items^a^	Factor 1
FDS	6. I am afraid of being thirsty to death.	0.881
	11. I am afraid of being penniless.	0.869
	10. I am afraid of a post-disaster epidemic.	0.860
	9. I am afraid of economic harm (damage to my house or the things in my house).	0.859
	1. I am afraid that the health of my first-degree relatives will be adversely affected (mother, father, and children).	0.857
	12. I am afraid that the health of my pets will be adversely affected.	0.833
	5. I am afraid that my own health will be adversely affected.	0.831

Eigenvalue		5.126
Variance explained		73.233
Variance cumulative		73.233
KMO		0.925 1476.178 p < 0.001
Bartlett's sphericity		

Abbreviations: FDS, Fear of Disasters Scale; KMO, Kaiser–Meyer–Olkin.

^a^Measurements of 265 participants in the first phase.

**Table 3:** Fit indices for the FDS.

Reference fit values^a^						
No	Index^b^	Weak fit	Acceptable fit	Perfect fit	Value of the study	Fit
1	*p* value		*p* > 0.05	*p* > 0.05	0.095	**Perfect fit**
2	χ²/df		Value < 5 Value < 3	Value < 3 Value < 2	1.51	**Perfect fit or acceptable fit**
3	RMSEA	Value < 0.10	Value < 0.08	Value < 0.05	0.059	**Acceptable fit**
4	RMR	Value < 0.10	Value < 0.08	Value < 0.05	0.076	**Acceptable fit**
5	SRMR	Value < 0.10	Value < 0.08 Value < 0.10	Value < 0.05	0.027	**Perfect fit**
6	GFI^c^	Value > 0.85	Value > 0.90	Value > 0.95	0.96	**Perfect fit**
7	AGFI^c^	Value > 0.80	Value > 0.90 Value > 0.85	Value > 0.95 Value > 0.90	0.92	**Acceptable fit or perfect fit**
8	NFI^c^	Value > 0.85 Value > 0.80	Value > 0.90	Value > 0.95	0.97	**Perfect fit**
9	NNFI^c^	Value > 0.85 Value > 0.80	Value > 0.90	Value > 0.95	0.98	**Perfect fit**
10	CFI^c^	Value > 0.85	Value > 0.90	Value > 0.95	0.99	**Perfect fit**
11	IFI^c^		Value > 0.90	Value > 0.95	0.99	**Perfect fit**
12	ECVI^c^		No fixed range, Smaller is better	No fixed range, Smaller is better	0.33	**Suitable**

*Note:* Bold emphasis in Table 3 shows whether the fit index value is suitable (perfect or acceptable).

Abbreviations: ECVI, expected cross validation index; FDS, Fear of Disasters Scale; GFI, goodness of fit index; IFI, incremental fit index; NFI, normed fit index; NNFI, non-NFI; RMR, root mean square residual; RMSEA, root mean square error of approximation; SRMR, standardized RMR.

^a^For reference fit values: [34–36].

^b^Measurements of 150 participants in the third phase.

^c^Gets a value between 0 and 1.

**Table 4:** Convergent validity of the FDS.

Sample^a^	FCS total score
FDS total score	
Correlation	0.310
* p* value	<0.001

Abbreviations: FCS, Fear of COVID-19 Scale; FDS, Fear of Disasters Scale.

^a^Measurements of 265 participants in the first phase.

**Table 5:** Item statistics of the FDS.

Items^a^	M ± SD	Item-total correlation
6. I am afraid of being thirsty to death.	3.10 ± 1.71	0.832
11. I am afraid of being penniless.	2.92 ± 1.67	0.805
10. I am afraid of a postdisaster epidemic.	3.20 ± 1.69	0.816
9. I am afraid of economic harm (damage to my house or the things in my house).	2.86 ± 1.62	0.771
1. I am afraid that the health of my first-degree relatives will be adversely affected (mother, father, and children).	3.64 ± 1.62	0.804
12. I am afraid that the health of my pets will be adversely affected.	3.22 ± 1.70	0.800
5. I am afraid that my own health will be adversely affected.	2.96 ± 1.70	0.768
Total score Cronbach's *α* = 0.939		

Abbreviations: FDS, Fear of Disasters Scale; SD, standard deviation.

^a^Measurements of 265 participants in the first phase.

## Data Availability

The data that support the findings of this study are available from the corresponding author upon reasonable request.

## Supporting Information

Supporting Information Fear of disasters is a psychological condition where an individual experiences intense fear or anxiety related to natural or man-made disasters such as earthquakes, floods, hurricanes, or even terrorist attacks. This fear can be triggered by past traumatic experiences, media exposure, or simply the anticipation of a potential disaster. Such fears often lead to heightened stress levels, difficulty in concentrating, and in severe cases, can result in phobias where the person may go to great lengths to avoid situations or places they associate with disasters. This can have a significant impact on their daily life and overall mental health. Fear of Disasters Scale is designed to assess the intensity and nature of fear related to various disasters, including both natural and man-made events. This study was conducted to develop a Fear of Disasters Scale and to determine its psychometric properties in a Turkish sample. As a result of the analyzes, Fear of Disasters Scale consisting of 7 items and one dimension was developed. The scale items and their Turkish and English meanings are given below. Item 1. Susuz kalmaktan korkarım (I am afraid of being thirsty to death). Item 2. Parasız kalmaktan korkarım (I am afraid of being penniless). Item 3. Afet sonrası salgından korkarım (I am afraid of a postdisaster epidemic). Item 4. Ekonomik zarardan korkarım (evim ya da evimdeki eşyaların zarar görmesinden; I am afraid of economic harm (damage to my house or the things in my house)). Item 5. Birinci derece akrabalarımın (anne, baba ve çocuk/çocuklar) sağlığı için korkarım (I am afraid that the health of my first-degree relatives will be adversely affected (mother, father, and children)). Item 6. Evcil hayvanımın sağlığı için korkarım (I am afraid that the health of my pets will be adversely affected). Item 7. Kendi sağlığım için korkarım (I am afraid that my own health will be adversely affected). Information about the scale (scale items, scoring instructions, etc.) is given in the supporting information section.
