# Supplementary material for: Developing the Fear of Disasters Scale and Exploring Its Psychometric Properties
Source: Depress Anxiety. 2024 Nov 25;2024:5565403. doi: 10.1155/2024/5565403 (PMC11919140; doi:10.1155/2024/5565403)
Supplement: Supporting Information — Fear of disasters is a psychological condition where an individual experiences intense fear or anxiety related to natural or man-made disasters such as earthquakes, floods, hurricanes, or even terrorist attacks. This fear can be triggered by past traumatic experiences, media exposure, or simply the anticipation of a potential disaster. Such fears often lead to heightened stress levels, difficulty in concentrating, and in severe cases, can result in phobias where the person may go to great lengths to avoid situations or places they associate with disasters. This can have a significant impact on their daily life and overall mental health. Fear of Disasters Scale is designed to assess the intensity and nature of fear related to various disasters, including both natural and man-made events. This study was conducted to develop a Fear of Disasters Scale and to determine its psychometric properties in a Turkish sample. As a result of the analyzes, Fear of Disasters Scale consisting of 7 items and one dimension was developed. The scale items and their Turkish and English meanings are given below. Item 1. Susuz kalmaktan korkarım (I am afraid of being thirsty to death). Item 2. Parasız kalmaktan korkarım (I am afraid of being penniless). Item 3. Afet sonrası salgından korkarım (I am afraid of a postdisaster epidemic). Item 4. Ekonomik zarardan korkarım (evim ya da evimdeki eşyaların zarar görmesinden; I am afraid of economic harm (damage to my house or the things in my house)). Item 5. Birinci derece akrabalarımın (anne, baba ve çocuk/çocuklar) sağlığı için korkarım (I am afraid that the health of my first-degree relatives will be adversely affected (mother, father, and children)). Item 6. Evcil hayvanımın sağlığı için korkarım (I am afraid that the health of my pets will be adversely affected). Item 7. Kendi sağlığım için korkarım (I am afraid that my own health will be adversely affected). Information about the scale (scale items, scoring instructions, etc.) is given in th [file 5565403.f1.docx]

**Fear Of Disasters Scale**

Dear participant, below are statements about you. Please read each item carefully and tick the option that best describes you. There is no right or wrong answer. What is expected from you is to help a scientific study by responding to the statements sincerely. Please express your views on all statements.

| Queue No | When responding to the items below, please consider the statement “WHEN THINKING OF DISASTERS” at the beginning of the items. | No fear | Negligible fear | Slight fear | Moderate fear | Considerable fear | Excessive fear |
| --- | --- | --- | --- | --- | --- | --- | --- |
| 1. | I am afraid of being thirsty to death. | 0 | 1 | 2 | 3 | 4 | 5 |
| 2 | I am afraid of being penniless. | 0 | 1 | 2 | 3 | 4 | 5 |
| 3 | I am afraid of a post-disaster epidemic. | 0 | 1 | 2 | 3 | 4 | 5 |
| 4 | I am afraid of economic harm (damage to my house or the things in my house). | 0 | 1 | 2 | 3 | 4 | 5 |
| 5 | I am afraid that the health of my first-degree relatives will be adversely affected (mother, father, and children). | 0 | 1 | 2 | 3 | 4 | 5 |
| 6 | I am afraid that the health of my pets will be adversely affected. | 0 | 1 | 2 | 3 | 4 | 5 |
| 7 | I am afraid that my own health will be adversely affected. | 0 | 1 | 2 | 3 | 4 | 5 |

**Scoring Instructions**

The FDS is a 7-item six-point Likert-type (0=No fear; 1=Negligible fear, 2=Slight fear, 3=Moderate fear, 4=Considerable fear, 5=Excessive fear) scale and has no reverse-coded item. The lowest and highest scores to be obtained on the FDS is 0 and 35, respectively. High scores on the scale indicate a high level of fear of disasters.
